# Leading Causes of Chronic Kidney Disease Among Dialysis Patients in Al-Baha Region, Saudi Arabia

**DOI:** 10.7759/cureus.49439

**Published:** 2023-11-26

**Authors:** Areej I Alhazmi, Abduljawad Hassan A Alghamdi, Khalid Abdulaziz M Alzahrani, Rizq Allah Abdullah B Alzahrani, Ibrahim Abdulaziz I Al Ghamdı, Mohammed Khamis B Alzahrani

**Affiliations:** 1 Internal Medicine/Nephrology, Faculty of Medicine, Al-Baha University, Al Baha, SAU; 2 Medicine, Faculty of Medicine, Al-Baha University, Al Baha, SAU

**Keywords:** chronic kidney disease, hypertension, diabetic nephropathy, esrd, dialysis

## Abstract

Introduction

Chronic kidney disease (CKD) and its progression to end-stage renal disease (ESRD) represent a growing health concern globally, with hypertension and diabetes commonly identified as primary etiological factors. This study evaluates the demographic and health profiles of individuals undergoing dialysis treatment in the Al-Baha region of Saudi Arabia, aiming to identify the predominant causes of ESRD and the associated socioeconomic and healthcare-related factors.

Methodology

This cross-sectional study analyzed data from patients receiving dialysis in Al-Baha. We assessed variables including etiology of ESRD, demographic data, presence of comorbid conditions, initial symptoms, and pre-dialysis health care engagement. Statistical analysis focused on the prevalence and correlations between the different variables and ESRD. The study also examined patients’ educational background and employment status to ascertain ESRD’s socioeconomic impact.

Results

The study found hypertension and diabetes as the leading causes of ESRD. Unknown etiologies accounted for 10.1% of cases, highlighting an area for further research. Notably, coronavirus disease 2019 (COVID-19) and cardiogenic shock emerged as potential new contributors, each representing 1.7% of cases. Most patients resided in urban areas, with the largest age group being 46 to 55 years. Men had a higher prevalence of ESRD than women. Low educational attainment was significant among patients, and unemployment due to ESRD was prevalent, pointing towards the need for vocational support. Clinical findings revealed late referrals to nephrologists, with a substantial proportion of diagnoses occurring in emergency settings. Family history suggested a higher-than-expected genetic component of CKD in the region.

Conclusion

The study confirms hypertension and diabetes as principal contributors to ESRD in Al-Baha while also pointing to the emergence of COVID-19 as a potential risk factor. Socioeconomic factors, including educational and employment status, are critical to patient management and outcomes. The high percentage of unknown etiologies and familial CKD prevalence warrants additional research. Improving early detection, enhancing patient education, and fostering timely nephrology consultations could mitigate the progression to ESRD and enhance patient quality of life.

## Introduction

Chronic kidney disease (CKD) is a widespread health concern characterized by gradually losing kidney function over time [1]. This complex disorder affects many individuals worldwide and is associated with significant morbidity, mortality, and healthcare costs [2]. CKD is classified into various stages based on kidney function, ultimately leading to end-stage renal disease (ESRD), which requires renal replacement therapies such as dialysis or kidney transplantation [3].

CKD stems from various sources, including modifiable and non-modifiable risk factors [4]. The causes of CKD vary among populations and regions, influenced by genetic, environmental, and socioeconomic factors [5]. However, common causes have been identified globally.

Diabetes is the primary cause of CKD worldwide, particularly type 2 diabetes, which is largely driven by sedentary lifestyles, unhealthy diets, and obesity [6-9]. Diabetic nephropathy, which is kidney damage resulting from diabetes, is characterized by glomerular damage and impaired kidney function [10]. Hypertension is another significant risk factor for CKD [11]. Persistent high blood pressure can damage kidney vessels, leading to decreased filtration of waste and disturbed fluid and electrolyte balance [12]. Other important causes include glomerulonephritis, an inflammatory condition of the glomeruli - the kidney’s filtering units - and polycystic kidney disease (PKD), a genetic disorder characterized by the formation of cysts in the kidneys [13]. Glomerulonephritis can result from various causes, including immune responses, infections, or genetic predisposition, while PKD is mainly due to genetic mutations [13].

Understanding the specific determinants of CKD within a particular region is crucial for implementing targeted interventions and preventative measures. This study aims to identify the primary factors contributing to CKD among dialysis patients in the Al-Baha region of Saudi Arabia, providing valuable information on the local epidemiology of CKD and supporting evidence-based strategies for prevention, early detection, and management.

## Materials and methods

We conducted a cross-sectional study with dialysis patients in the Al-Baha region of Saudi Arabia. The research took place across four dialysis centers in Al Baha, Mandaq, Mikwah, and Aqiq to ensure diverse representation from the region. Eligible participants were those diagnosed with ESRD, currently receiving dialysis treatment, and willing to engage in the study. We excluded patients with cognitive impairments or those unable to give informed consent.

For the sample size, we used the Raosoft sample size calculator. Using 5% as a margin of error, 95% as a confidence interval, and 50% as response distribution, we assume that 119 patients would be adequate. 

Trained research assistants administered a structured questionnaire to collect data. The questionnaire captured demographics, the primary cause of kidney disease, and health-related information. We inquired about the initial type of dialysis received, the time and place of ESRD diagnosis, attendance at nephrology clinics, and whether they underwent an ultrasound or biopsy before dialysis. We also collected information on pre-diagnosis symptoms, pre-dialysis medications, exposure to contrast agents, and any family history of ESRD.

Data analysis

We performed descriptive statistical analyses, calculating frequencies, percentages, means, and standard deviations for participant demographics and health characteristics. The analysis was conducted using IBM SPSS Statistics for Windows, Version 26.0. (IBM Corp., Armonk, NY, USA), with significant findings (p<0.05) reported.

Ethical considerations

The Al Baha University’s institutional review board granted ethical approval (Approval No. REC/MED//BU-FM/2022/61) on December 27, 2022. All participants provided informed consent, and we ensured confidentiality and voluntary participation. Our study complied with the Declaration of Helsinki, respecting participant privacy and rights.

## Results

We analyzed the demographic characteristics of 119 dialysis patients in the Al-Baha region. Most participants (62.2%) were from the Al Baha Center, with the remaining from the Mandaq Center (8.4%), Mikwah Center (27.7%), and Aqiq Center (1.7%). The largest age group was 46 to 55 years, representing 25.2% of the participants. Men comprised 58.8% of the cohort. Regarding education, 28.6% had no formal education, and 21.8% had completed more than secondary education. Half of the participants (50.4%) were unemployed, while 16.0% were governmental employees. We observed statistically significant variations in the distribution of participants by center, age group, gender, education level, employment status, and monthly income (p<0.05; Table 1).

**Table 1:** Participant demographic data (N=119) Abbreviation: SAR, Saudi Riyal.

Demographic Data		N	Percentage	P-value
Center	Al Baha Center	74	62.2%	P < 0.0001
	Mandaq Center	10	8.4%	
	Mikwah Center	33	27.7%	
	Aqiq Center	2	1.7%	
Age in Years	18-25	3	2.5%	P < 0.0001
	26-35	10	8.4%	
	36-45	22	18.5%	
	46-55	30	25.2%	
	56-65	22	18.5%	
	66-75	23	19.3%	
	76-85	9	7.6%	
Gender	Male	70	58.8%	P = 0.05
	Female	49	41.2%	
Educational Level	Illiterate	34	28.6%	P = 0.04
	Primary School	21	17.6%	
	Middle school	13	10.9%	
	Secondary school	25	21.0%	
	>Secondary school	26	21.8%	
Employment	No work	60	50.4%	P < 0.0001
	Students	4	3.4%	
	Governmental employee	19	16.0%	
	private sector employee	7	5.9%	
	Retired	29	24.4%	
Monthly Income	Less than 5000 SAR	73	61.3%	P < 0.0001
	5000 to 10000 SAR	30	25.2%	
	10001 - 15000 SAR	13	10.9%	
	15001- 20000 SAR	3	2.5%	

**Figure 1:**
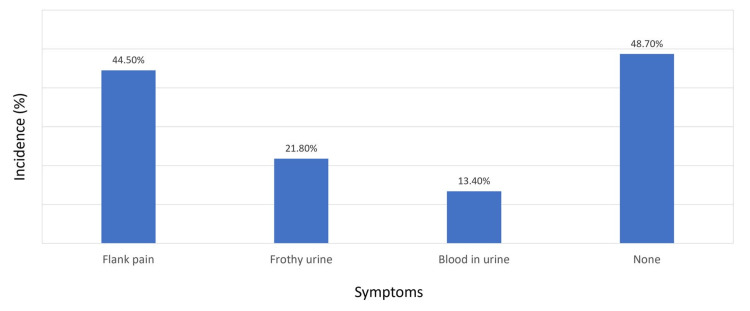
Incidence of symptoms prior to dialysis

In assessing the health characteristics of the same 119 patients, we found that 86.7% had been living with diabetes for over a decade. Hemodialysis was the chosen modality for 93.3% of the patients. Nearly half (47.9%) had been receiving dialysis for more than three years. The diagnosis of chronic kidney disease was made less than six months before starting dialysis for 22.7% of patients, and 23.5% received their diagnosis in an emergency setting. Tertiary hospitals were where 68.1% of patients received their ESRD diagnosis. Most (62.2%) had attended a nephrology clinic before starting dialysis. Ultrasound had been performed for 91.6% of patients, whereas only 25.2% had undergone a renal biopsy before initiating dialysis. Notably, 20.2% had a relative with ESRD, with siblings being the most common (52.2%; Table 2). Figure 1 shows that 44.5% of patients experienced flank pain, 21.8% noticed frothy urine, 13.4% saw blood in their urine, and 48.7% reported none of these symptoms before beginning dialysis.

**Table 2:** Participant health characteristics and medical histories (N=119)

Participant Health Data		N	Percentage
When were you diagnosed with diabetes?	2-5 years	1	3.3%
	6-10 years	3	10.0%
	>10 years ago	26	86.7%
What was your first dialysis modality?	Hemodialysis	111	93.3%
	Peritoneal dialysis	8	6.7%
When did you start on dialysis?	< 6 months ago	22	18.5%
	6 months to 1 year	14	11.8%
	1 to 3 years	26	21.8%
	>3 years ago	57	47.9%
When was your diagnosis with end-stage renal disease?	< 6 months before starting dialysis	27	22.7%
	6 months to 1 year before starting dialysis	19	16.0%
	>1 year before starting dialysis	28	23.5%
	Sudden in the clinic and referred to dialysis	17	14.3%
	Sudden in emergency and referred to dialysis	28	23.5%
Where was your diagnosis of end-stage renal disease?	Emergency room	7	5.9%
	Peripheral hospital	31	26.1%
	Tertiary hospital	81	68.1%
Did you follow up in the nephrology clinic before dialysis?	No	45	37.8%
	Yes	74	62.2%
If Yes, for how long?	<6 months	7	12.1%
	6 months to 1 year	9	15.5%
	1 to 3 years	20	34.5%
	>3 years	22	37.9%
Did you have an ultrasound done before dialysis?	No	10	8.4%
	Yes	109	91.6%
Did you have a renal biopsy done before dialysis?	No	89	74.8%
	Yes	30	25.2%
Have you done any imaging with contrast within the last 6 months?	No	94	79.0%
	Yes	25	21.0%
Does anyone in your family have end stage renal disease?	No	95	79.8%
	Yes	24	20.2%
If Yes, who?	Father	5	21.7%
	Mother	4	17.4%
	Grandparents	3	13.0%
	Sibling	12	52.2%
	Son/Daughter	3	13.0%

**Figure 2:**
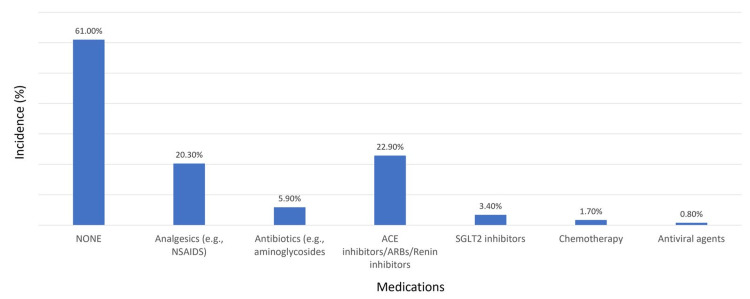
Incidence of medication use six months before starting dialysis Abbreviations: NSAID, nonsteroidal antiinflammatory drug; ACE, angiotensin-converting enzyme; SGLT2, sodium-glucose cotransporter-2.

Figure 2 shows that 61.0% of patients did not take any nephrotoxic medications six months before starting dialysis. Of those who did, 20.3% used analgesics like nonsteroidal anti-inflammatory drugs, 5.9% took antibiotics such as aminoglycosides, 22.9% were on angiotensin-converting enzyme inhibitors or angiotensin receptor blockers or renin inhibitors, 3.4% were prescribed sodium-glucose cotransporter-2 inhibitors, 1.7% underwent chemotherapy, and 0.8% took antiviral medications.

**Figure 3:**
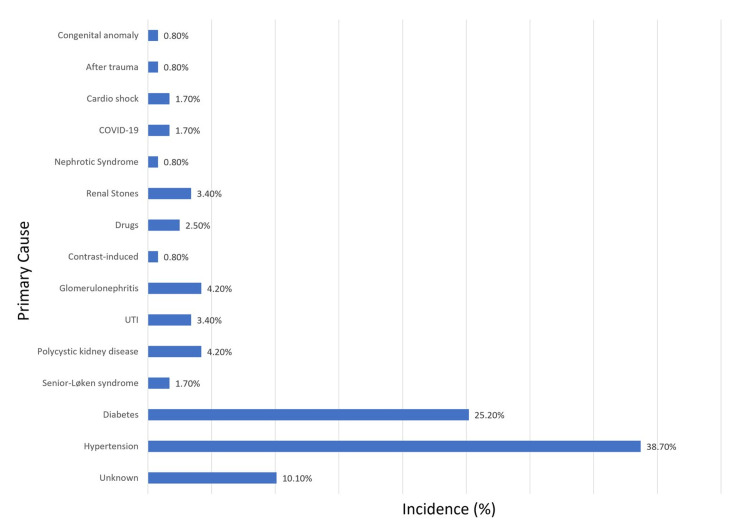
Primary causes of kidney disease among study participants Abbreviations: COVID-19, coronavirus disease 2019; UTI, urinary tract infection.

According to Figure 3, hypertension was the leading cause of kidney disease, responsible for 38.7% of cases, followed by diabetes (25.2%). The cause was unidentified for 10.1% of the patients, with other causes including senior-Løken syndrome (1.7%), polycystic kidney disease (4.2%), urinary tract infections (3.4%), glomerulonephritis (4.2%), contrast media exposure (0.8%), medication-related issues (2.5%), renal calculi (3.4%), nephrotic syndrome (0.8%), coronavirus disease 2019 (COVID-19; 1.7%), cardiogenic shock (1.7%), trauma (0.8%), and congenital anomalies (0.8%). 

## Discussion

This study provides a detailed examination of dialysis patients’ demographic and health characteristics in Al-Baha, Saudi Arabia, offering valuable insights that augment our understanding of the region-specific burden of ESRD. Our investigation confirms that hypertension and diabetes are the predominant etiologies of ESRD, corroborating previous studies that identify these conditions as leading causes of chronic renal failure both regionally and globally [4,13-19].

The high prevalence of diabetes as a contributing factor aligns with the well-documented diabetes epidemic in Saudi Arabia [16]. The global health community acknowledges the pressing risks posed by uncontrolled diabetes and hypertension, significantly accelerating the progression to ESRD [4]. In our cohort, a concerning 10.1% of ESRD cases had unknown causes, echoing findings from other studies and suggesting potential genetic, viral, or environmental factors warranting further exploration [20-23].

Our findings also introduce COVID-19 and cardiogenic shock, each accounting for 1.7% of ESRD cases, as emerging contributors to renal failure. These novel etiologies call for more robust longitudinal studies to elucidate their long-term renal impacts and pathophysiological mechanisms [22,23].

Demographically, the centralization of dialysis services in urban areas was anticipated, reflecting Al-Baha’s population distribution. Notably, the predominance of middle-aged adults (ages 46 to 55 years) undergoing dialysis mirrors global trends [24,25], and the higher incidence of ESRD in men in our study is consistent with global patterns [26]. These demographic insights are critical for targeted interventions and resource allocation within the healthcare system.

The educational profile of our patients, with a significant segment being illiterate, underscores the nexus between educational attainment and health literacy determinants of chronic disease management efficacy [27]. This highlights the urgent need for patient education strategies that accommodate varying literacy levels, potentially through visual aids and multimedia resources.

Economically, the high unemployment rate among our participants sheds light on the substantial economic burden of dialysis, which can preclude gainful employment. Addressing this through vocational rehabilitation could be beneficial for improving the economic stability of ESRD patients. Clinically, this region’s widespread preference for hemodialysis aligns with national patterns [28]. However, the tendency for late referrals to nephrology services is troubling, as nearly one in four patients were diagnosed in emergency settings. This suggests a gap in early detection and nephrology involvement, highlighting the need for improved screening and management protocols to slow disease progression.

The pre-dialysis healthcare journey revealed that while most patients had consulted a nephrologist and undergone ultrasonography, a smaller fraction had a renal biopsy. This discrepancy may reflect patient or clinician reservations about invasive procedures and warrants further investigation into the decision-making processes in pre-dialysis care.

Our study identified a familial occurrence of ESRD in 20.2% of patients, a figure significantly higher than reported elsewhere [29,30]. This suggests a potential genetic predisposition within the population, emphasizing the need for genetic counseling and investigation into hereditary renal diseases. Symptomatically, the presence of nonspecific symptoms like flank pain, frothy urine, or hematuria was documented. Yet, nearly half of the patients reported no such symptoms, consistent with the silent onset of chronic kidney disease reported in the literature [13]. This reinforces the importance of vigilant symptom assessment in at-risk populations.

This study has several limitations that should be acknowledged. Firstly, the sample size is relatively small and geographically limited to the Al-Baha region, which may not comprehensively represent the dialysis population in Saudi Arabia or the Middle East. Secondly, its cross-sectional design does not allow for the establishment of causality between observed health characteristics and outcomes. Thirdly, while the reliance on self-reported data may introduce recall bias, particularly concerning the disease progression timeline and previous medical interventions, this was partially mitigated by our methodological approach. In our study, we focused on a single region with a small sample size, which enabled us to conduct in-depth interviews and review medical records to verify the accuracy of the responses, thereby aiming to reduce recall bias and add strength to our findings. Lastly, the absence of a control group of non-dialysis patients with chronic kidney disease limits the generalizability of the findings to the broader ESRD population. These limitations suggest that the results should be interpreted cautiously and that further longitudinal and multi-center studies are necessary to validate and extend these findings.

## Conclusions

The present study identifies hypertension and diabetes as the primary causes of CKD in the Al-Baha region, consistent with global trends. The significant family history suggests the potential for inherited kidney diseases. The data also reveal critical gaps in early diagnosis and management, pointing to the need for improved pre-dialysis care to slow the progression of kidney disease. The proportion of patients with ESRD of unknown etiology indicates an area ripe for future research to determine contributing factors.
